# Abstracts and Gleanings

**Published:** 1882-10-20

**Authors:** 


### ABSTRACTS AND GLEANINGS.

**Foreign Bodies in the Air Passages.**—This paper was statistical, and contained generalizations from over one thousand cases which he had collected. The conclusions to which he had been led differed from those of most text-books, but were founded upon a careful comparison of the results obtained by prompt surgical interference and 'those which followed spontaneous expulsion: His conclusions were:

1. When a foreign body is lodged either in the larynx, trachea or bronchia, the use of emetics, errhines, or similar means, should not be employed, as they increase the sufferings of the patient and do not increase his chances of recovery.

2. Inversion of the body and succussion, though sometimes useful, are dangerous; and should not be practiced unless the windpipe has been previously opened.

3. The presence simply of a foreign body in the larynx, trachea, or bronchia, does not make bronchotomy necessary.

4. While a foreign body causes no dangerous symptoms, bronchotomy should not be performed.

5. While a foreign body remains fixed in the trachea or bronchia, as a general rule, bronchotomy should not be practiced.

6. When symptoms of suffocation are present, or occur at frequent intervals, bronchotomy should be resorted to without delay.

7. When the foreign body is lodged in the larynx, there being no paroxysms of strangulation, but an increasing difficulty of respiration from œdema or inflammation, bronchotomy is demanded.

8. When the body is movable in the trachea, and excites frequent attacks of strangulation, bronchotomy should be performed.

Dr. Mears told of a case where he had been removing a tongue, and an assistant let a sponge, which was not firmly enough attached to a probang, drop into the patient's larynx. He opened the trachea at once—not by dissection, but by plunging the knife boldly into the trachea. He also narrated some particulars of a case which had been under treatment for serious pulmonary disease. The boy was one day swinging, when he fell out upon his face on the ground, where a penny was found which he had then expelled from his lung. After this all the symptoms gradually disappeared and the patient got well. He also told of a case in which a distinguished surgeon operated, and the instant the trachea was opened a coin was expelled from the wound.—*Dr. Weist in Amer. Surg. Association.*

**The Rational and Routine Treatment of Venereal Disease.**—Dr. Post asked if the reader would include excision of the primary lesion with the cautery.

Dr. Greenough answered yes.

Dr. Post mentioned that of the large number of cases seen by him at Chelsea, the character of the primary sore was almost always masked by the use of "blue-stone," which is so universal among seamen.

Dr. Langmaid expressed his surprise at the sweeping strictures of the reader on eaustics, for, while agreeing in the main, his own experience had given him great faith in the application of nitric acid and acid nitrate of mercury. He believed he secured better results than with iodoform, and avoided the disagreeable smell of the latter, often a matter of great importance. The pain from the application of the acid nitrate of mercury was at first severe, but could be very much controlled by the preliminary application of carbolic acid.

In regard to the so-called secondary and tertiary ulcerations of the mouth and throat, Dr. Langmaid dissented from Dr. Greenough. He found in these cases great good to result from the occasional application of acid nitrate of mercury, together with constitutional treatment. He had seen the most obstinate ulcers heal repeatedly under this method of treatment, and even the perforations of the hard palate, which are usually considered as hopeless.

In regard to the question of marriage, Dr. Greenough said that while he could not conceive of any combination of circumstances which would justify a physician in guaranteeing a patient who had had syphilis from the possibility of future relapses, he did think that in cases where treatment had been kept up for two years, and another year had elapsed without any relapse, the chances were decidedly against any manifestations of a specific nature either in the patient or his progeny. He thought that the number of cases of patients who, having had an attack of syphilis, went through life without ever hearing from it again, must be greater than formerly. Where, on the other hand, a patient has had relapses, he never can feel safe as to the future.

Dr. Parks asked if Dr. Greenough believed in a sympathetic gonorrhœal disease of the eyes that is not due to infection. The reader had never seen such a case, but it was described by older writers.

Dr. Waterman asked if Dr. Greenough could give any statistics as to the spontaneous cure of syphilis.

The reader could not, but thought there must be quite a large number, judging from the very slight amount of treatment that many cases receive. In dispensary practice a large proportion of patients cease their attendance as soon as their symptoms have disappeared. He had known of cases where, from recklessness or carelessness, practically no systematic treatment had been followed out, and yet the disease seems to have been entirely eradicated.—*Boston Med. and Surg. Journal.*

**The Value of Pure Codeine.**—In an article published in the *Journal de Therapeutique*, Dr. Leblanc says: The reason why authorities differ as to the merits of codeine is probably due to the fact that it is frequently adulterated by admixture of chlorhydrate of morphia. Out of 100 samples of so-called codeine syrup, recently examined, 23 were found not to contain a particle of this substance. As far as my observations go, I have found codeine to be a reliable soporific, valuable because of its mildness and the facility with which its effects are dispelled without leaving any unpleasant results. These effects are especially notable in cases of acute and subacute bronchitis, so much so that I am inclined to believe this alkaloid possesses an elective sedative action on the mucous membranes of the bronchi and larynx. Under its influence the tickling sensations in the larynx, which induce coughing are speedily calmed, and the patient falls into a quiet and refreshing sleep. Another advantage in favor ot codeine is, that it is well tolerated by those persons who, owing to nervous temperaments, or from some unknown idiosyncrasy, are unable to take morphine.

There are a great many patients who, as it were, react against the effects of opium or morphia, either by experiencing nausea, or from a natural tendency to struggle against the hypnotic effects of morphia, as soon as they begin to be felt.

In such cases pure codeine gives most excellent results; its effects are progressively soothing and culmiuate in a quiet sleep, unattended by either giddiness or those strange sensations, amounting often to delirium, which frequently accompany morphia.

From the foregoing observations, it would seem that codeine is a valuable remedy as a sedative in insomnia, colds, bronchitis, asthma and whooping-cough, provided it is perfectly pure.—*Med. and Surg. Reporter.*

**Can a Man have Syphilis Twice.**—The British Medical Journal reports the following valuable remarks by Mr. Jonathan Hutchinson, on this interesting topic:

The man whom we have just seen offers a remarkable example of the occurrence of a second chancre soon after the first. His second sore has been, as I have repeatedly demonstrated, characteristically indurated. He is quite candid, and makes no doubt that this sore was the result of contagion. Yet it is barely a year since he had his first chancre, and this was followed by an eruption, of which he had scarcely got clear when this second sore occurred. The case is proof that a man may may have an indurated sore on the penis within a year of a former one, but it is not proof that he may have syphilis twice, for this patient has not as yet had any constitutional symptoms as the result of the last chancre. If, however, you ask me for an answer to the general question, Can a man have true, complete syphilis twice? then I must reply clearly that he can. Such cases are rare—as rare, perhaps, as examples of second attacks of smallpox—but they do occur. I am at present attending a gentleman who has a terrible phagedenic chancre and rupial eruption, and who unquestionably had complete syphilis, chancre, sore-throat and rash seven years ago. I have also a second case nnder care, very much milder, but illustrating exactly the same fact, with almost precisely similar dates. Second chancres are, however, far more common than second attacks of constitutional syphilis. Many of them are the result of fresh contagion, but seem to have no power to produce constitutional symptoms; but others are not from contagion at all, but form in connection with a taint still remaining from the first attack. It is a most important fact that indurations may form in the penis, in every respect exactly like Hunterian chancres, not distinguishable in any way, and yet they may be merely recurred sores, and the products of constitutional taint. I have seen this over and over again; and M. Alfred Fournier, of the St. Louis Hospital, has written a very instructive paper on this form of sore. In the case of our patient, it is obviously impossible to say, after the statement which I have just made, whether or not his present sore is the result of fresh contagion. It may simply be a relapse, or it may be a gumma. He, however, confesses to exposure; and, as the sore followed in due course, it is probably true that he was afresh inoculated. Second attacks of syphilis are sometimes, as in the case just mentioned, very severe. The same has, I believe, been occasionally noted in recurred attacks of variola. As a rule, however, they are mild, or even abortive. Third attacks may even occur; and so may, as we are told, third attacks of smallpox. We must explain such facts, I expect, by reference to individual peculiarity and idiosyncrasy, but it is important that they should be known. The belief that syphilis can occur but once in a lifetime is very widely spread among a certain class of the public. I have watched with amusement the change in expression in many a young gentleman's face when he got my reply to his smiling suggestion—"A man cannot, I suppose, have the disease a second time.—*Med. and Surg. Rep.*

**Physiological Effects of Prolonged Bathing.**—In an investigation on the above subject, published in Paris Medical, for December, and giving a very accurate account of the effects which baths produce on the system, according to their duration and temperature, Dr. Thery has arrived at a number of conclusions which are both interesting and true. He says: A bath at 97° Fahrenheit is without effect on the circulation. All baths below 97° reduce the action of the heart. The beats, however, acquire greater energy. The pulse retains perfect regularity. Circulation is not reduced in direct ratio with the temperature of the water, but it is influenced by the duration of the bath.

When baths of 75° or less are prolonged for an hour, arterial pulsation continues decreasing after exit from the water. Baths at or below the temperature of the body quicken circulation. This acceleration is proportional to the temperature of the water. The pulse is irregular and the heart fluttering.

Baths between 97° and 99° are without effect on animal heat. Baths below 97° reduce the temperature of the body. Baths between 92° and 97° cause a loss of 0.97° to 1.46°; this reduction is obtained within half an hour; after this the thermometer remains stationary, even should the bath be continued for two hours.

In baths at 86° or under the fall in temperature is more gradual; it is in proportion to the duration of the bath.

The first effect of a bath at 72° or less causes a slight elevation of temperature. The fall in temperature obtained by means of a half hour bath at 93° is almost equal to that produced by a bath at 72° continued for an hour. After a bath above 82°, continued for an hour or two, temperature has an upward tendency, although for the following twelve hours it remains from 0.5 to 1° below what it was before the bath. After a bath under 81°, the thermometer continues falling during the next twenty minutes following exit. During the twelve hours following a prolonged bath, at from 64° to 81°, the thermometer indicates a reduction of 1° to 1.50° from the initial temperature.

All baths at or above the temperature of the body produce a rise in central temperature. The rise, in proportion to the temperature of the water, is progressive. A bath at 108°, continued for nineteen minutes, raises the temperature of the body to 104°. A bath at 68° progressively raised to 95° produces a fall in temperature, A bath at 97° gradually reduced to 75° causes, as a first effect, a fall in temperature; but, subsequently, in proportion as the temperature of the bath decreases, that of the body rises. It is only between 91° and 97° that baths can be continued for a long time without causing suffering.

In water, the sensation of cold acts by reflex action, first on the smooth muscular fibres, and later on the striated.

Hot baths predispose to syncope; they are followed by profuse perspiration.

All baths, when long continued, are debilitating.—*Med. and Surg. Reporter*.

**Shall the Travelling Mesmerizer be Abolished.**—A new and curious medico-legal question has recently been raised in the Swiss courts, an account of which is given in the Correspondenz-Blatt.

In 1880–81 a famous "magnetizer," Donato, traveled through Switzerland giving exhibitions. One of the results was the development of a furor for mesmerizing each other, especially among the young people. In July, 1881, a young girl applied for admission to the Maternity Hospital at Berne, saying that she was pregnant. She stated further that, being visited by a young man one evening, he mesmerized her and then violated her person. She was delivered of a child in September. Her story reached the ears of the "juge d'instruction" of Berne. He caused the matter to be investigated. Dr. Ladame, of Neuchatel, was appointed to investigate the matter. He did so, and gave a very elaborate report thereon. The question is, he says, an entirely new one in medical jurisprudence. There exist only four cases reported in medical literature.

These four cases are cited at length by Dr. Ladame, with the opinions of experts given upon them at the time. In one case the plea was asserted to be a fraud, because the woman was able to give a fnll account of the affair. This, in all cases, it was agreed, showed that the state produced could not have been a hypnotic one.

The other cases showed that violation could take place during "nervous sleep" without the knowledge and against the desire of the woman.

This opinion, sustained by Tardieu, Brouardel and others, is one which would naturally be drawn from the known characteristics of this peculiar condition; and it may become a matter of importance in the future that this fact be known to medical men.

The question will at once follow whether traveling mesmerizers should not be forbidden to exercise their arts. The influence which they exert upon the health of their subjects is certainly not good. Should it become known that mesmerizing is a simple thing, and that a certain per cent. of young women are susceptible to the hypnotic condition, bad results to morals might follow.

On the other hand, the plea that violation was done by the help of mesmeric practices will almost always be difficult to prove. Dr. Brouardel, indeed, asserts that since the researches of Charcot it is possible to distinguish absolutely the hypnotic condition from simulation. This view is not, however, as yet generally accepted.

We trust that the subject may continue as rare and novel as it is now, though this can hardly be expected.—*N. Y. Med. Record.*

**Treatment of Bone Felon.**—While looking over The Druggists Circular of a late date, my attention was directed to an article headed "Bone Felon," in which the writer has offered a remedy for its cure, and which, he states, was copied from the London Lancet. The remedy is as follows: As soon as the disease makes its appearance, apply to the spot a fly blister, about the size of your thumb nail, and let it remain for six hours, at the expiration of which time, directly under the surface of the blister, may be seen the felon, which can be instantly taken out with the point of a needle or lancet.

Permit me here to state, that while I consider the London Lancet a most valuable work of its kind, and ganerally excellent authority on which to base our opinions respecting the nature and mode of treating diseases, yet I am free to confess that I have but little or no faith in the remedy which it offers for the cure of bone felon.

Any one who has studied carefully the history or nature of this complaint, or has had much experience in the treatment of it, knows full well that the simple application of a fly plaster to the part affected will not cure it.

This disease is not, as many suppose, simply a swelling of the soft parts of the finger, or a collection of matter under the skin, but a disease more deeply seated—a disease of the bone and periosteum, or the membrane surrounding the bone and giving nourishment and vitality to it.

When this bone or its membrane is seriously injured from any cause whatever, inflammation is set up, the membrane as well as the bone becomes morbidly sensitive, matter collects under it, and by its continued accumulation, presses upon and distends the part, giving rise to the excruciating pain which is always experienced in this disease.

Admitting this to be the true character of the complaint, what is the remedy to be applied? It is a very simple one, and which any person with the least nerve can readily and quickly perform. With a sharp knife or razor lay the part freely open to the bone.

This will give exit to the matter, and by relieving the distention of the diseased parts will afford instant relief.

All that now remains to be done is to apply a slippery elm, flax-seed, or bread and milk poultice. The addition of a little lead water to the poultice will assist in allaying the excitement in the parts and add materially to its curative effects.—*Druggistss Circular.*

**Case of Pregnancy in a Woman at the Age of Sixty-two.** —Cases of pregnancy occurring in women who have passed the half century, and more especially in one who has borne over a score of children, are undoubtedly of extreme rarity.

Early on the morning of 29th November, 1880, Dr. W. John Kennedy, of Dalkeith (Edinburgh Medical Journal, June, 1882), was summoned to attend Mrs. M., residing in Back street, Dalkeith. Patient was sixty-two years of age, her catamenia had generally been regular (the last occurring in the middle of February), and she was in her twenty-third pregnancy. On arrival, Dr. K. found labor going on naturally, os well dilated, pains strong and regular, and the head presenting. Having waited a short time, and finding that the expulsive powers showed no signs of rupturing the membranes, he punctured them, after which four or five pains sufficed to expel the child.

Naturally, the first question which arises in one's mind is, are the data on which this woman's age is set down at sixty-two correct? In regard to this, Dr. K. calls attention to the following points in evidence:

1st. The statements made to him by the woman herself. These were given freely and explicitly, and the most careful cross-examination entirely failed in producing any contradictions. Besides, there was no possible object to be gained by the woman fabricating such a remarkable history as that given by her.

2d. The fact that Mrs. M. was a nurse under Dr. K. in 1870, when she said that she was over fifty; and certainly she had quite the appearance of a woman of that age.

3d. The number of confinements which she had had at term, namely, twenty (one twin), with the addition of three miscarriages.

4th. The fact that in June, 1879, when her third husband applied to the parochial board at Selkirk for relief, he stated his wife's age to be sixty years, i. e., sixty-one in the following October.

From a medico-legal point of view, Dr. K. ventures to think that this case presents some features of special interest. Whilst it is impossible to lay down a hard and fast rule as to the age beyond which a woman may not conceive and bear a child, still every well-authenticated case of pregnancy at an advanced age is of value, as showing how late in life a woman may have, and actually has had, a child. It may be said roughly that the child-bearing age in woman ceases between forty and fifty; but as impregnation may take place at exceptionally early periods of life, so it may also occur at ages considerably beyond fifty. In a table given in

Taylor's Medical Jurisprudence (1865, p. 876), there are twelve cases quoted at ages from fifty to fifty-four. In the same work there is also a case mentioned in which a healthy woman bore a child at the age of sixty, menstruation having continued up to that time. Two others at sixty-three and sixty-five are noted, but their authenticity is doubted. He also gives two cases, recorded by Haller, in which women at sixty-three and seventy bore children.—*Amer. Jour. Ob.*

**The Scientific Principles of Inhalation.**—In the British Medical Journal, Dr. Robert J. Lee thus writes:

The important relation recently shown to exist between septic agents diffused in the atmosphere and certain forms of pulmonary disease, is receiving so much attention that it is well to consider the scientific principles on which it depends. Experiments show that it is possible to diffuse antiseptic agents in the atmosphere by evaporation, and that organic substances may be preserved in such atmosphere without decomposition; or, in other words, that the air may be treated as a fluid, and be charged with antiseptics which prevent bacterial development. Now, when we burn any of the hydrocarbons or gum-resins, we do not volatilize them, and the air is rendered antiseptic, except to the extent that a certain amount escapes unburnt and is diffused. It follows, from this, that destruction of the antiseptic agent must be avoided. After numerous experiments—and the general results of those made a few years ago were presented at the Cambridge meeting of our Association—it appears that carbolic acid is the only antiseptic, as far as I know, which can be volatilized in a definite and constant manner. This is a most important fact in treatment, and deserving attention. If a solution of 1 part of carbolic acid in 80 of water be distilled under slight pressure, the vapor will contain the same proportion of the acid as the solution during the process of boiling; so that we can obtain vapor of any strength and diffuse it in the atmosphere. Other antiseptics are either more or less volatile; as, for example, thymol, which comes off very rapidly from the boiling water, as does also benzoic acid: so that they are not convenient for inhaling.

It is also necessary to observe that vaporizing a solution in the form of spray does not volatilize the antiseptic to any great extent, since the dew settles quickly on the nearest surfaces, and does not rise and diffuse itself as the vapor of steam does.

Again, the sprinkling of solutions on clothes does not necessarily secure diffusion of the agent; for, at the ordinary temperature, the agent may not evaporate, but will remain in the texture of the cloth. There are other details which will occur to those who reflect on these matters, and will secure success as may fairly be expected from the scientific use of atmospheric disinfectants. I trust that I shall be forgiven for egotism in saying that I think the small inhaler exhibited by Maw, Son & Thompson for me, at the International Sanitary Exhibition at the present time, affords the most convenient and scientific means of atmospheric disinfection; and that, until more perfect methods are offered to the profession, I believe it will be found deserving of more general attention than it has yet received.—*Med. and Surg. Reporter.*

**Tonsillotomy by Ignipuncture.**—The British Medical Journal, after commenting on the dangers of removing hypertrophied tonsils by all of the old methods, says: "It is now alleged that with the thermo-cautery this serious accident (hemorrhage) is no longer to be dreaded. M. Krishaber, who has tried it during two years, and has collected more than forty cases ("Annales des Maladies de l'Oreille et du Larynx") has never had any accident after this treatment, and the results obtained have been lasting. It is likewise a novel application of a method which he has found perfectly successful for granulations of the larynx and pharynx. He proceeds as follows: The patient is placed—firmly, if a child—as if for laryngoscopic examination, in front of the operator, the mouth open, the tongue held back by a large spatula, the bottom of the throat well illuminated. M. Krishaber generally uses Paquelin's narrow-pointed thermo-cautery, heated to red heat. When it is only required to modify the nutrition of the gland, he gives preference to Trouve's polyscopic galvano-cautery. The puncture of the gland, made as deeply as possible with the point of the instrument, should be repeated five or six times at each sitting. An interval of two or three days is left between the sittings, so as to allow the fall of the eschar, and to estimate the result. The operation is not at all painful, and pain from burning is rarely felt. Nothing need be administered after the operation except, in some cases, a gargle of warm water, slightly carbolized.—*Med. and Surg. Rep.*

**Swallowing a Baby.**—Dr. J R. Harwell, M. D., reports in Nashville Medical Journal (Nashville, Tenn.) the case of a little child who swallowed a small china doll: "It was about an inch in length, by about three lines in thickness, with legs extended, and arms flexed at the elbow, so as to be at right angles with the baby. The arms measured from the elbow to the end of the hand about four lines. When the doll slipped into the pharynx there was an involuntary spasmodic effort to expel it, and, no doubt, it was arrested in the œsophagus for a few moments before passing through the cardiac orifice, as, in addition to indications of impending suffocation, the little patient complained for a short time of acute pain in the region of the stomach. This, however, soon passed off and she fell into a quiet slumber, in which condition I found her. On awaking she complained of no pain, and, assuring the frightened young ladies that, in my humble opinion, the danger was passed, I left. The next morning the doll was found in the dejections of the child, having traversed the entire alimentary canal. This, of course, gave inexpressible relief to the mind of the anxious mother, who carefully laid the doll away to become a family relic, to be exhibited in the years to come, on account of its associations and wonderful adventures. Though asked to do something to rid the stomach of the doll, I declined to do so, believing it to be innocuous, and that it would easily pass through the intestinal tract without ill effect. Emetics, with the view of expelling the foreign body, would have been highly improper, and even dangerous, as the irregular shape of the doll made it liable to lodge in the œsophageal tract."

**The Feeding of Infants.**—This subject was di eussed at the one hundred and twelfth annual meeting of the Medical Society of the State of New Jersey, at Spring Lake, New Jersey, May 28 and 29, 1878. Special Report for the Medical Record, Vol. 13, No. 23. (Extract from the report.) "Answers to this question differed with the residence of the physician—the conntry and city manifesting each its peculiar needs. The mother's milk is generally conceded to be the best for the infant, but even when this is abundant, it may be disadvantageous for the individual living on it, and a substitute be necessary. Country practitioners recommend cow's milk as the best substitute, while many in cities and towns speak highly of condensed milk. Of the preparations so various and so highly commended by those who put them on the market, the Imperial Granum seems to hold the first place in the estimation of medical observers. All agree in condemning the use of nursing-tubes as unclean, even with the best of care."

**Can Dreams be Controlled?**—The Lancet says that a French investigator, M. Delaunay, finds, from experiments upon himself, that the character of his dreaming may be controlled by stimulating various portions of the brain by means of heat. By covering his forehead with a layer of wadding he gets sane, intelligent dreams. He has also experimented on modes of lying, which favor the flow of blood to particular parts, increasing their nutrition and functional activity. He has observed that the dreams he has while lying upon his back are sensorial, variegated, luxurious. Those experienced when on the right side are mobile, full of exaggeration, absurd, and refer to old matters; but those produced when on the left side are intelligent and reasonable, and relate to recent matters; in these dreams one often speaks. These observations may be correct, so far as M. Delaunay is concerned, but most people who lie on their back, especially after eating, are apt to find their dreams anything but luxurious.—*Med. and Surg. Reporter, July 15, 1882.*

**Treatment of Facial Erysipelas by Scarifications and Opium.**—Dr. V. Netzetzky, of Russia, describes (Vracheb. Vedom, No. 11, 1882) a treatment of erysipelas successfully practiced by native barbers, and consisting in numerous superficial vertical scarifications made by a razor over the whole diseased part. After the bleeding spontaneously stops, they moisten the affected surface by an aqueous solution of opium (two grains to the drachm), and cover with a layer of cotton wool. Rapid cure follows, as the author alleges; he himself observed two cases successfully treated in this way.—*N. Y. Med. Record.*

**Recent Advances in Urinary Analysis.**—Among the more or less recent additions to our knowledge of urinary analysis, Lobisch (Wien. med. Woch., Dec. 10, 17, 1881) gives the following as the more important: 1. The absorption of carbolic acid in large (toxic) quantities is marked by the disappearance from the urine of the sulphates which furnish a precipitate with barium chloride, the reason being that most of the sulphur compounds have entered into combination with the phenol, and in this state are not acted upon by this precipitating agent. 2. The presence of an abnormal quantity of indican in the urine has been noted in certain wasting affections, as well as in peritonitis and intestinal obstruction. 3. Peptonuria occurs chiefly in connection with supperative processes on any part of the body, and in croupous pneumonia. 4. Chyluria is found in fatty degeneration of the urinary tract, in malignant tumors, especially of the pancreas, in phosphorus poisoning, acute yellow atrophy of the liver, fat embolism, etc. —*N. Y. Med. Jour.*

**Simple Treatment of Excessive Sweating.**—Dr. T. H. Currie, of Lebanon, says, in the *Michigan Med. News:* "For over thirty years I have used the following prescription, without a single failure, in sweats from whatever cause: "Alcohol, O. j; sulphate of quinine, ʒ j. Wet a small sponge with it and bathe the body and limbs, a small surface at a time, care being taken not to expose the body to a draft of air in doing it. In one case a neighboring physician was poisoned while dressing a mortified finger. He suffered untold misery and was drenched with perspiration for a number of days, and his life despaired of. When I saw him, I ordered him to be bathed immediately in the above solution, and that this be repeated once in two hours. The third application stopped all perspiration, and convalescence began at once."—*N. Y. Med. Record.*

**Comparative Size of Drops.**—The following table approximately gives the average number of drops in a fluid drachm of the various classes of U. S. P. preparations:

| | | | |
|---|---|---|---|
| Sulphuric ether | 174 | Mixtures | 80 |
| Fluid extracts | 141 | Vinegars | 77 |
| Spirits | 141 | Sy'p not containing f. exts. | 69 |
| Tinctures | 136 | Solutions (1 exception) | 66 |
| Volatile oils | 131 | Diluted acids | 61 |
| Oleo-resins | 124 | EXCEPTIONS. | |
| Acids (3 exceptions) | 123 | Sol. nitrate of mercury | 131 |
| Wines | 106 | Nitromuriatic acid | 76 |
| Fixed oils | 103 | Muriatic acid | 70 |
| Sy'ps containing fl. exts. | 07 | Sulphurious acid | 159 |

—*Exchange.*

THE Alienist and Neurologist cites the case of a family in which there were three idiotic boys. One of these was so unfortunate as to accidentally receive such a blow on his head as had the effect of causing sufficient change in his mental condition as to enable his parents afterward to make a lawyer out of him.

**Codeia in Diabetes.**—In the British Medical Journal, Dr. R. Shingleton Smith relates some cases of diabetes treated by codeia, in which this drug produced very good effects. It has a remarkable power of checking the elimination of sugar, and a corresponding improvement in the health of the patient results. It would appear that alkalies and all other methods of treatment are far inferior to the treatment by codeia, which may be considered to have almost a specific action on the disease. The facts seem to justify decided language with regard to the use of codeia, which should not be permissive, but imperative, in all cases of advanced diabetes mellitus; whatever else may be given, codeia should first be given, and in fairly large doses, until some physiological effect is produced. Even dieting appears to sink into insignificance, alongside of codeia, and in one case this drug alone was sufficient without any dieting, the patient being on a mixed diet all the time.—*Amer. Med. Weekly.*

**Hydrophobia Cured.**—The treatment consisted in giving one part of potassium bromide (60 to 120 grains) a day, with syrup of codeine, chloral, and in addition to this a subcutaneous injection of nitrate of pilocarpine, repeated 3 to 4 times a day at first, but afterwards only twice. Under the influence of this treatment, the crisis was little removed; dysphagia diminished, then ceased; agitation disappeared, the appetite returned, and at thc end of fifteen days the cure was considered complete.

**Detection of Vesical Calculi in Children.**—Volkmann suggests the following: The bladder is to be nearly empty, and under the influence of anesthetic, an examination is to be made with the left hand in the rectum. The right hand presses firmly above the symphisis pubis, forcing the bladder down upon the rectum. In this way even small calculi can be detected, although he has generally found them to be larger when extracted than he had expected on examination.—*N. C. Med. Jour.*

**Hydrate of Chloral and Tinct. Iodine.**—According to the authority of Pavesi, the therapeutic powers of tincture of Iodine are increased by the addition of cnloral hydrate, which dissolves in it without decomposition, and is readily miscible with water without precipitation. This combination possesses remarkable hemostatic virtues, from its marked coagulating powers over albumen.—*Pacific Medical Journal.*

**An Irish Hermaphrodite.**—Two Irishmen, having been rather intimate with a certain young woman, were compelled by other members of the family to make provision for the prospective offspring. If it turned out to be a boy, by mutual agreement, Pat was to take care of it; if a girl, that duty was to devolve on Mike. After several months of suspense, Mike came one day running towards Pat, his face beaming with a smile of the utmost satisfaction. "Hello, Pat, the bloody thing has come!" "Well," says Pat, "what is it, a boy?" "No." "What, then, a girl?" "No, it's a d—d naygur."—*Obst. Rev.*

**Disinfection of Urine.**—In the Medical Annals, Dr. F. C. Curtis states, hydrate of chloral has the property of disinfecting urine. In August last he received a specimen of urine, four ounces, containing five grains of chloral to the ounce. No special care was taken to facilitate its preservation. It has been simply corked, and has been several times opened. It is now perfectly transparent, of a clear amber color, and has a fresh, urinous, slightly balsamic odor. A slight semi-flocculent deposit covers the bottom of the vial. The chemical analysis is identical with the notes made when it was recent: sp. gr. 1.015. acid, albumen one-fourth. The preservation of the albumen is a marked test. On microscopic examination, the epithelial cells are as perfect as in recent urine. No casts were noted and none are now seen. The specimen seems to be quite the same as when freshly voided, and is manifestly instructive and of considerable interest.—*Med. and Surg. Rep.*

**Ataxy and Sewing Machines.**—In the Union Medicale, M. Octave Guelliot contributes a valuable paper on two cases of locomotor ataxy in women employed on sewing machines. In hysterical women, working at the sewing machine seems to be, in certain cases, the occasional cause of the appearance of locomotor ataxy. The symptoms commence in the lower limbs and progress upward. Shooting pains traverse the limbs from below upward. Improvement is noticed when the patient rests, and it may last a long time. Working at the machine by means of a treadle probably acts chiefly by the concussion, which is diffused throughout the spinal cord. Therefore, the continuous movement of the treadle is dangerous to the work-woman, and endeavors should be made to substitute some other motor for the foot-power.—*Med. nnd Surg. Rep.*

**American Medical Association.**—The Secretaay of the Surgical Section of the American Medical Association requests all gentlemen who are writing papers to be read before that section at its next meeting to notify him at least one month before the time of meeting. And also to give at the same time the titles of their papers and their probable length. By so doing the titles will bd published in time for the members to be prepared for the proper discussion of the various subjects. And he suggests that all gentlemen taking part in the debates reduce their remarks to writing, so that the report may be more accurate and valuable as a mirror of present scientific thought. Communications should be addressed to WM. A. BYRD, M.D., Secretary Surgical Section, American Medical Association, 407 Jersey street, Quincy, Illinois. —*Med. and Surg. Rep.*

**A New Cinchona Alkaloid.**—Several English Chemists have found in Flueckiger's China cuprea an alkaloid which, although it in several respects closely resembles quinia, must nevertheless be considered a new one. It has provisionally been called cupreine. The Cuprea cinchona forms vast forests in Columbia, and has been exported since 1871. It varies very much in value, some specimens containing no quinine, others up to two per cent.—*New Idea.*

